# Association of mitochondrial DNA variation with high myopia in a Han Chinese population

**DOI:** 10.1007/s00438-023-02036-y

**Published:** 2023-06-05

**Authors:** Shilai Xing, Siyi Jiang, Siyu Wang, Peng Lin, Haojun Sun, Hui Peng, Jiaying Yang, Hengte Kong, Sheng Wang, Qingshi Bai, Ruowen Qiu, Wei Dai, Jian Yuan, Yunlong Ma, Myopia Associated Genetics and Intervention Consortium, Xiaoguang Yu, Yinghao Yao, Jianzhong Su

**Affiliations:** 1 School of Ophthalmology and Optometry and Eye Hospital, Wenzhou Medical University, Wenzhou, 325027 China; 2 Wenzhou Institute, University of Chinese Academy of Sciences, Wenzhou, 325011 China; 3 National Clinical Research Center for Ocular Disease, Wenzhou, 325027 China; 4 Institute of PSI Genomics, Wenzhou, 325024 China; 5 Oujiang Laboratory, Zhejiang Lab for Regenerative Medicine, Vision and Brain Health, Wenzhou, 325101 Zhejiang China

**Keywords:** High myopia, Mitochondrial genomics, Susceptibility, Haplogroup, Oxidative phosphorylation

## Abstract

High myopia (HM), which is characterized by oxidative stress, is one of the leading causes of visual impairment and blindness across the world. Family and population genetic studies have uncovered nuclear-genome variants in proteins functioned in the mitochondria. However, whether mitochondrial DNA mutations are involved in HM remains unexplored. Here, we performed the first large-scale whole-mitochondrial genome study in 9613 HM cases and 9606 control subjects of Han Chinese ancestry for identifying HM-associated mitochondrial variants. The single-variant association analysis identified nine novel genetic variants associated with HM reaching the entire mitochondrial wide significance level, including rs370378529 in *ND2* with an odds ratio (OR) of 5.25. Interestingly, eight out of nine variants were predominantly located in related sub-haplogroups, i.e. m.5261G > A in B4b1c, m.12280A > G in G2a4, m.7912G > A in D4a3b, m.94G > A in D4e1, m.14857 T > C in D4e3, m.14280A > G in D5a2, m.16272A > G in G2a4, m.8718A > G in M71 and F1a3, indicating that the sub-haplogroup background can increase the susceptible risk for high myopia. The polygenic risk score analysis of the target and validation cohorts indicated a high accuracy for predicting HM with mtDNA variants (AUC = 0.641). Cumulatively, our findings highlight the critical roles of mitochondrial variants in untangling the genetic etiology of HM.

**Supplementary Information:**

The online version contains supplementary material available at 10.1007/s00438-023-02036-y.

## Introduction

The 16,569-bp human mitochondrial (mt) genome is circularly organized with 37 genes encoding 13 proteins, 22 transfer RNAs and 2 ribosomal RNAs that are essential for oxidative phosphorylation (OXPHOS) and production of cellular energy in the form of ATP (Schon et al. 2012). Being maternally inherited, mitochondria genomes have acquired sequential accumulation of single-nucleotide variants (mtSNVs), which combined to define genealogical haplogroups (Yao et al. 2002; van Oven and Kayser 2009; Wei et al. 2019). Many mtSNVs directly affect mitochondrial function to influence eye related manifestations, including visual loss from the optic nerve and retinal disease, visual field loss from retrochiasmal visual pathway damage, and ptosis and ocular dysmotility from extraocular muscle involvement (Schrier and Falk 2011; Yu-Wai-Man and Newman 2017). Since both nuclear DNA (ntDNA) and mitochondrial DNA (mtDNA) encode proteins essential for mitochondrial function, defects in either of the nuclear or mitochondrial genome cause mitochondrial dysfunction. The biological mechanisms of dysfunction include impaired mitochondrial energy production, oxidative stress, mitochondrial DNA instability, abnormalities in the regulation of mitochondrial dynamics and mitochondrial quality control, and disturbed cellular interorganellar communication (Gorman et al. 2016; Yu-Wai-Man and Newman 2017). Mutations affecting mitochondrial function have been reported to determine inherited eye disease including Leber hereditary optic neuropathy (LHON) as well as other disease listed in RetNet (https://sph.uth.edu/retnet/disease.htm), and are also being increasingly implicated to be associated with age-related eye disorders, including diabetic retinopathy, age-related macular degeneration, glaucoma, cataract, and keratoconus (Jones et al. 2007; Udar et al. 2009; Izzotti et al. 2010; Schrier and Falk 2011; Bek 2017; Miller et al. 2020; Xu et al. 2021b). Unveiling the genetic components of mitochondrial disease have not only improved genetic diagnosis, but also provided important insights into the pathophysiologic basis of these disorders and potential therapeutic targets (Yu-Wai-Man and Newman 2017).

High myopia (HM) defined as myopia with a spherical equivalent refractive error of less than or equal to − 6.0 diopters (D) has the global prevalence of 4.0% and especially great prevalence in East Asian populations (4.5%–21.6%)(Baird et al. 2020; Tan et al. 2021; Xu et al. 2021a). HM is featured by progressive eyeball axial length longer than 26 mm with practically irreversible damage to the retina. It frequently accompanies the increased risk of serious ocular complications, like most notably retinal degeneration and even detachment, which can cause severe vision impairment or blindness (Saw et al. 2005; Morgan et al. 2012). Mitochondrial oxidative stress, as demonstrated in other retinal and macular diseases with atrophy of retinal pigment epithelium (RPE) and choroids, was implicated in myopia and HM occurrence and development (Francisco et al. 2015; Yang et al. 2022).

A growing number of mutations in nuclear genes encoding mitochondrial proteins were reported to be associated with HM. For example, a premature stop codon mutation c.157C > T (p. Gln53*) co-segregating with HM within SCO2, which encodes for a copper homeostasis protein involved in mitochondrial cytochrome c oxidase activity, was discovered by exome sequencing in 4 individuals from a 11-member family of European descent from the United States (Tran-Viet et al. 2013). Wang et al. (2017) reported a novel missense mutation: c.798C > G (p. Asp266Glu) in *NDUFAF7*, co-segregating with pathogenic myopia in a 11-member Chinese family. This mutation caused amino acid substitution D266E, which impaired mitochondrial complex I activity to decrease ATP levels in cultured cells (Wang et al. 2017). Further, one linkage mapping study identified and replicated two myopia susceptibility loci *MFN1* and *PSARL* in UK twin subjects (Andrew et al. 2008), and both genes interact with OPA1 to regulate mitochondrial fusion and the inhibition of mitochondrial-led apoptosis, respectively. Besides, genome-wide association studies (GWAS) detected two associated loci, *MIPEP* (the mitochondrial intermediate peptide gene, MIM 602241) in HM of Han Chinese population (Shi et al. 2011), and *BLID* in pathogenic myopia of Japanese population (Nakanishi et al. 2009). The MIPEP protein is primarily involved in the maturation of oxidative phosphorylation (OXPHOS) related proteins; the product of this gene performs the final step in processing a specific class of nuclear-encoded proteins targeted to the mitochondrial matrix or inner membrane. Given that the retina is the most energy consuming tissue in the eye and MIPEP is involved in the process of energy generation via oxidative metabolism, MIPEP is more likely to play a vital role in normal eye development (Shi et al. 2011). BLID harboring the BH3-like domain plays a pro-apoptotic role by inducing a caspase-dependent mitochondrial cell death pathway (Nakanishi et al. 2009).

Although those studies have shown the genetic evidences of mitochondrial dysfunction involved in myopia or HM occurrence, no mitochondrial germline variant has been reported to be pathogenic or a susceptibility loci associated with myopia or HM. In order to explore the putative mitochondrial loci involving in HM risk, we conducted the first large-scale case–control cohort study of whole mitochondria-wide association among 9613 HM cases and 9606 controls (aged from 6 to 18) from the Han Chinese population (Su et al. 2023).

## Materials and methods

### Ethics approval and patient recruitment

The present study was approved by the Ethics Committee of the Wenzhou Medical University Affiliated Eye Hospital. Written informed consent conforming to the tenets of the Declaration of Helsinki was obtained from all participating individuals or their guardians before the study. Totally, 21,227 Chinese schoolchildren aged from 6 to 18 were recruited from the Myopia Associated Genetics and Intervention Consortium (MAGIC) at the Eye Hospital of Wenzhou Medical University (Zhejiang Eye Hospital) through the Institute of Biomedical Big Data (Xu et al. 2021a). 48% of the study participants were females. HM was defined as a spherical equivalent refraction (SER) of (sphere + [cylinder/2]) of -6 diopters (D) or less.

### Mapping and calling

The whole exome sequencing (WES) data (captured by the Twist mitochondrial panel as a spike-in with Twist’s human core exome) of 9613 HM cases and 9606 controls obtained after quality control of nuclear genome data (Su et al. 2023) were aligned to rCRS (NC_012920.1) as mitochondrial reference genome sequence using the Burrows–Wheeler Aligner (BWA 0.7.12) (Li and Durbin 2009), and Sambamba 0.6.6 (https://lomereiter.github.io/sambamba/) was used for sorting and marking duplicates. The unpaired reads were removed using Samtools 1.9, and the discordant or split reads were removed using Samblaster 0.1.26. The combination of GATK 4.0.11.0 commands: BaseRecalibrator, ApplyBQSR, HaplotypeCaller with the parameter ‘–sample-ploidy 1’, CombineGVCFs and GenotypeGVCFs were used for calling single-nucleotide variants (SNVs) and insertions/deletions (InDels) (Van der Auwera et al. 2013).

### Sample QC, haplogroup assignment and variant annotation

The coverage and average depth for each individual were calculated by Bamdst 1.0.9 (https://github.com/shiquan/bamdst). The number of variants for each individual, site sequencing depth and allele frequency in each cohort were calculated by vcftools 0.1.17 (Danecek et al. 2011). Outliers (> 3 SD from the mean) of the average depth and number of variants for each individual within each cohort were further discarded. Haplogroup annotation was performed using standalone haplogrep2.2.9 (Weissensteiner et al. 2016). Detailed variant annotation were obtained using the online tool, MSeqDR (https://mseqdr.org/mvtool.php). The LHON variants (https://www.mitomap.org/foswiki/bin/view/MITOMAP/MutationsLHON) were checked in the annotation results.

### PCA, match by PCA and PC correlation

The principle component analysis (PCA) for each haplogroup was performed in PLINK 2.0 (Purcell et al. 2007) and GCTA 1.93 (Yang et al. 2011) using bi-allele single-nucleotide polymorphisms of mitochondria genome with MAF > 5%. Within each haplogroup, we set one case matching one control on the basis of the top three PCs by PCAmatchR to reduce potential bias in mitochondrial population structure (Brown et al. 2021)(Figs. S2, S3). The paired samples were pooled together to do PCA for mtDNA, ntDNA and mcDNA (1136 nuclear genes with mitochondrial protein localization in MitoCarta3) with MAF > 5%, respectively. The correlation of PCs was calculated and plotted by Corrplot 0.92 (https://cran.r-project.org/web/packages/corrplot/index.html).

### Mitochondria-wide single-variant association analysis

Totally, 4,904 bi-allele SNPs with minor count larger than 2 among 8,797 cases and 8,797 controls were tested for three GWAS models including plink_firth, SingleScore and SingleWald. The firth regression analyses for the binary traits in HM (cases as 2 and controls as 1) were performed using PLINK 2.0 with the adjustment for sex, the top 20 PCs as covariates. Meanwhile, single variant Wald and Score test were performed using RVTESTS (Zhan et al. 2016) with the adjustment for sex, the top 20 PCs as covariates.

### Structure analysis of protein

The structural impact of ND5 and ND6 variants was investigated using a human cryo-EM structure of complex I (PDB:5XTD) (Guo et al. 2017). The structures of ND5 and ND6 were colored through the labeled l and m chains in 5XTD. The protein structure visualization was performed using UCSF Chimera v.1.16 (Pettersen et al. 2004).

### Polygenic risk score analysis

The paired samples were randomly split into 7:3 of target and validation cohorts. The single sites of mitochondria-wide association analysis in the target cohort were performed using plink firth method. Then polygenic risk scores (PRS) were calculated by PRSice-2 (Choi and O’Reilly 2019) for individuals in the validation cohort using summary results for single nucleotide polymorphisms (SNPs) obtained from above target cohort GWAS accounting for linkage disequilibrium (LD) pruning to exclude SNPs that were correlated (*r*^2^ > 0.1) with another variant within a 250 kb window under different *P*-value thresholds. The AUC plot of PRS was analyzed and displayed by R package pROC (Robin et al. 2011).

### Expression analysis across tissues

The gene-level reads per kilobase million mapped reads (RPKM) values of the Genotype-Tissue Expression (GTEx) RNA-seq data (https://www.gtexportal.org/home/) were used across 55 tissue types, including peripheral retina samples (Consortium 2013). The gene expression distribution in tissues was displayed in violin plots using R package “vioplot”.

## Results

### MtDNA variants landscape and haplogroup assignment

**Fig. 1:**
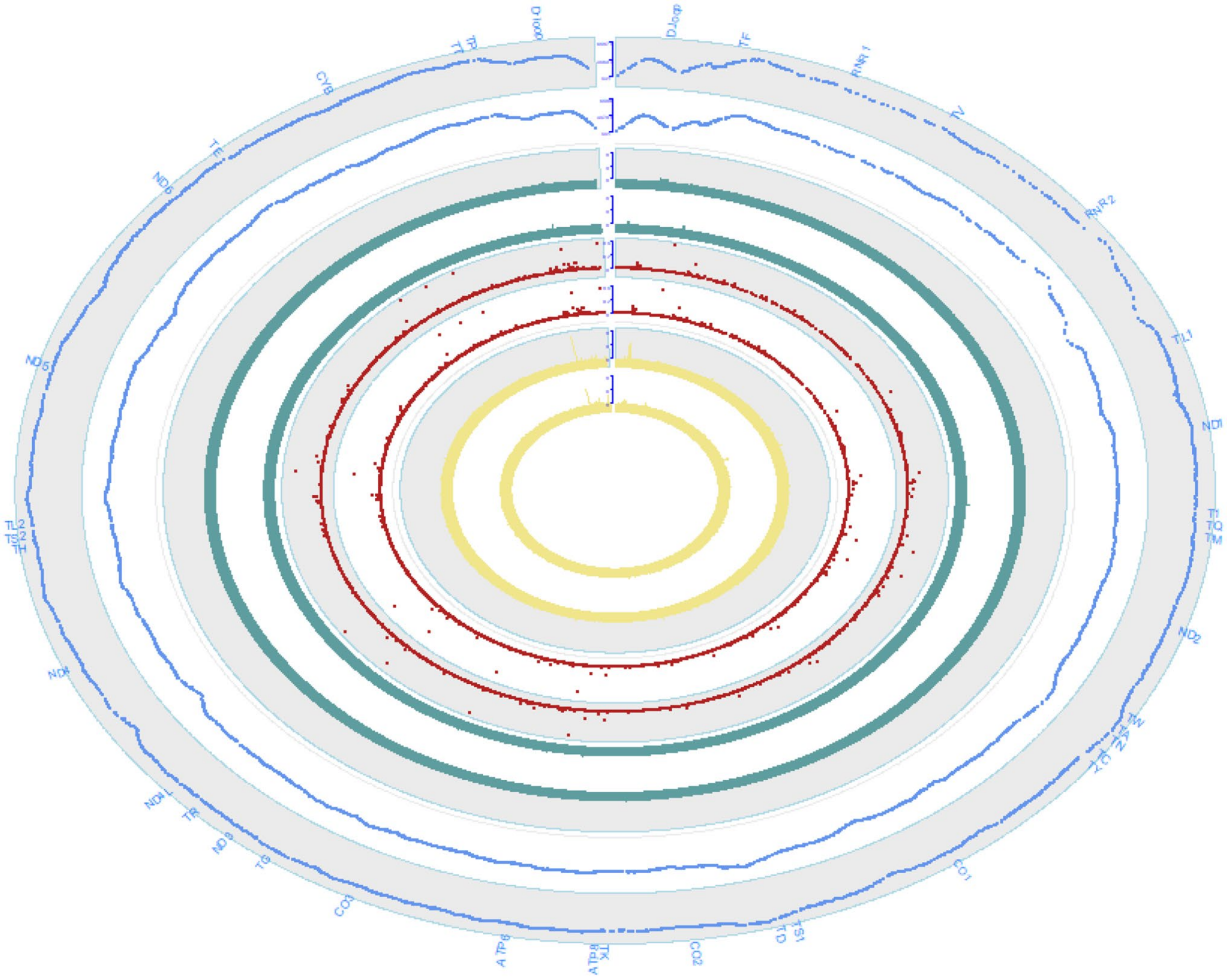
The landscape of mtSNV features in HM and the control, including mean sequencing depth, missing rate, minor allele frequency and heteroplasmy rate for each variant. The outer space indicated the gene positions. From outer to inner, the first and second rings showed mean sequencing depth of sites in HM and controls with axis scale from 0 to 6500 X; the third and fourth rings showed missing rate of sites in HM and controls with axis scale from 0 to 0.002; the fifth and sixth rings showed minor allele frequency of sites in HM and controls with axis scale from 0 to 0.49; the seventh and eighth rings showed heteroplasmy rate of sites in HM and controls with axis scale from 0 to 0.009

A total of 9613 cases and 9606 controls were enrolled for mitochondria-wide genetic association analysis according to the whole exome wide study from the Myopia Associated Genetics and Intervention Consortium (MAGIC) project (Su et al. 2023). After removing reads related to nuclear mitochondrial DNA segments (nuMTs), the uniquely mapped reads to the mitochondrial genome were used for further analysis. We eliminated 132 cases and 39 controls identified as outliers of sequencing depth and calling variants in the cohort (Supplementary Material, Fig. S1). Due to the highly saturated sequencing depth of whole mitochondrial (5065 X for cases and 4513 X for controls), the number of calling variants showed no correlation with sequencing depth in HMs and controls (Supplementary Material, Fig. S1). About 99.9% of the mitochondrial genome was sequenced with a mean depth of at least 1000 X. A total of 5,099 mtDNA variants were annotated by MSeqDR (Falk et al. 2015), including 3712 (72.8%) in the homoplasmic state, 35 (0.7%) in the heteroplasmic state, and 1352 (26.5%) in ‘both’ states. Sixteen major mitochondrial mutations were annotated to be relevant with ophthalmologic manifestations (Table 1). Eleven variants including m.11778G > A, m.14502T > C, m.4025C > T, m.9804G > A, m.10237T > C, m.11253T > C, m.11696G > A, m.12811T > C, m.14279G > A, m.14325T > C, and m.14831G > A were related to LHON (Leber’s Hereditary Optic Neuropathy); three variants including m.3243A > G, m.1640A > G, and m.3995A > G were related to MELAS (Mitochondrial encephalomyopathy, Lactic acidosis, and stroke-like episodes); and two variants including m.13528A > G and m.8597 T > C were related to Leigh Syndrome. The MITOMAP confirmed LHON mutation (m.11778G > A) and MELAS mutation (m.3243A > C) showed no difference of allele frequency between HMs and controls. Two missense variants, m.14325 T > C in ND6 and m.14831G > A in Cytb, were significantly enriched in HM with the homoplasmic state (Table 1). Individuals were assigned into haplogroups with mean quality score of 0.81 by the Haplogrep2 (Weissensteiner et al. 2016). After filtering down haplogroups with fewer than 20 individuals, ancestry-match analysis in each haplogroup was performed for 11 haplogroups using PCAmatch (Brown et al. 2021), resulting in the identification of 8797 pairs (Supplementary Material, Fig. S2). The first three principal components (PCs) of mtDNA variants were plotted to show the well-matched HMs and controls for 8797 pairs (Supplementary Material, Fig. S3). The site sequencing depth, site missing rate, site frequency and the heteroplasmy ratio for cases and controls, calculated using 4,936 mtDNA variants including 32 InDels, exhibited similar trends for each parameter between HMs and controls in the variant landscape (Fig. 1). For the 4904 mtSNPs, we found that 4526 (92.3%) and 4475 (91.3%) variants have been reported in the MITOMAP and gnomAD databases, respectively. Similar results were reported in the mtDNA variation database of 3241 Chinese individuals called MTCards (Wang et al. 2022), where 3595 (92.4%) and 3566 (91.6%) variants were found in the MITOMAP and gnomAD databases, respectively.

**Table 1:** Major mitochondrial mutations with ophthalmologic manifestations annotated by MSeqDR

Variant	AAΔ	Gene	HM (*n* = 9469)	Control (*n* = 9549)	*P*_fisher (het^a^; hom^b^)	MITOMAP_disease	MITOMAP_status^c^	ClinVar^d^	MTCards^e^	MITOMAP^f^
m.11778G > A	R340H	ND4	3 (het); 3 (hom)	1 (het); 3 (hom)	0.37;1	LHON/Progressive Dystonia	Cfrm	Pathogenic	–	–
m.14502T > C	I58V	ND6	196 (hom)	1 (het); 192 (hom)	1;0.80	LHON	Reported -possibly synergistic	Benign	0.019 (hom)	0.0036
m.4025C > T	T240M	ND1	12 (hom)	1 (het); 31 (hom)	1;0.0054	Putative LHON	NA	Benign	0.0025 (hom); 0.00030 (het)	0.0080
m.9804G > A	A200T	CO3	1 (het); 27 (hom)	1 (het); 36 (hom)	1;0.31	LHON/MS	Reported	Conflicting interpretations of pathogenicity	0.0040 (hom)	0.0029
m.10237T > C	I60T	ND3	2 (hom)	4 (hom)	0.69 (hom)	LHON	Reported	Benign	–	0.0017
m.11253T > C	I165T	ND4	13 (hom)	21 (hom)	0.23 (hom)	LHON/PD	Reported	Benign	0.0015 (hom); 0.0003 (het)	0.0050
m.11696G > A	V312I	ND4	1 (het); 183 (hom)	205 (hom)	0.50;0.31	LHON/LDYT/DEAF/hypertension	Reported-possibly synergistic	Benign	0.015 (hom)	0.0063
m.12811T > C	Y159H	ND5	580 (hom)	520 (hom)	0.047 (hom)	Possible LHON factor	Reported	Conflicting interpretations of pathogenicity	0.071 (hom); 0.0015 (het)	0.012
m.14279G > A	S132L	ND6	2 (het)	1 (het)	0.62 (het)	LHON	Reported	Uncertain significance	0.00031 (het)	0.00012
m.14325T > C	N117D	ND6	60 (hom)	20 (hom)	5.0E−06 (hom)	LHON	Reported	Benign	0.0028 (hom)	0.0010
m.14831G > A	A29T	CytB	1 (het); 53 (hom)	1 (het); 23 (hom)	1;0.00050	LHON	Reported	Benign	0.0012 (hom)	0.0020
m.3243A > G	NA	tRNA-Leu	9 (het)	14 (het)	0.40 (het)	MELAS/Leigh Syndrome/DMDF/MIDD/SNHL/CPEO/MM/FSGS/ASD/Cardiac + multi-organ dysfunction	Cfrm	Pathogenic/Likely pathogenic	–	–
m.1640A > G	NA	tRNA-Val	5 (hom)	5 (hom)	1 (hom)	MELAS	Reported	Likely benign	–	–
m.3995A > G	N230S	ND1	6 (hom)	1 (het)	1;0.015	MELAS	Reported	Uncertain significance	–	0.0003
m.13528A > G	T398A	ND5	1 (hom)	7 (hom)	0.070 (hom)	LHON-like/LHON/MELAS	Reported	Conflicting interpretations of pathogenicity	0.00031 (hom)	0.0011
m.8597T > C	I24T	ATP6	2 (hom)	1 (het); 2 (hom)	1;1	Leigh Syndrome	Reported	Likely benign	–	0.0003

^a^Het, heroplasmy

^b^Hom, homoplasmy

^c^MITOMAP’s variant categories regarding disease involvement include “Cfrm” where multiple independent laboratories and/or researchers have evaluated the variant and reported compelling functional evidence of its pathogenicity, “reported” where a variant has been seen in affected individuals and is considered to be related to disease without rigorous validation, and “Benign” status where a variant is not independently validated in MITOMAP at this time

^d^ClinVar, the clinical significance reported in ClinVar

^e^MTCards, allele frequency in MTCards database

^f^MITOMAP, allele frequency in MITOMAP database

### No correlation between mtDNA and nuclear genetic structure

**Fig. 2:**
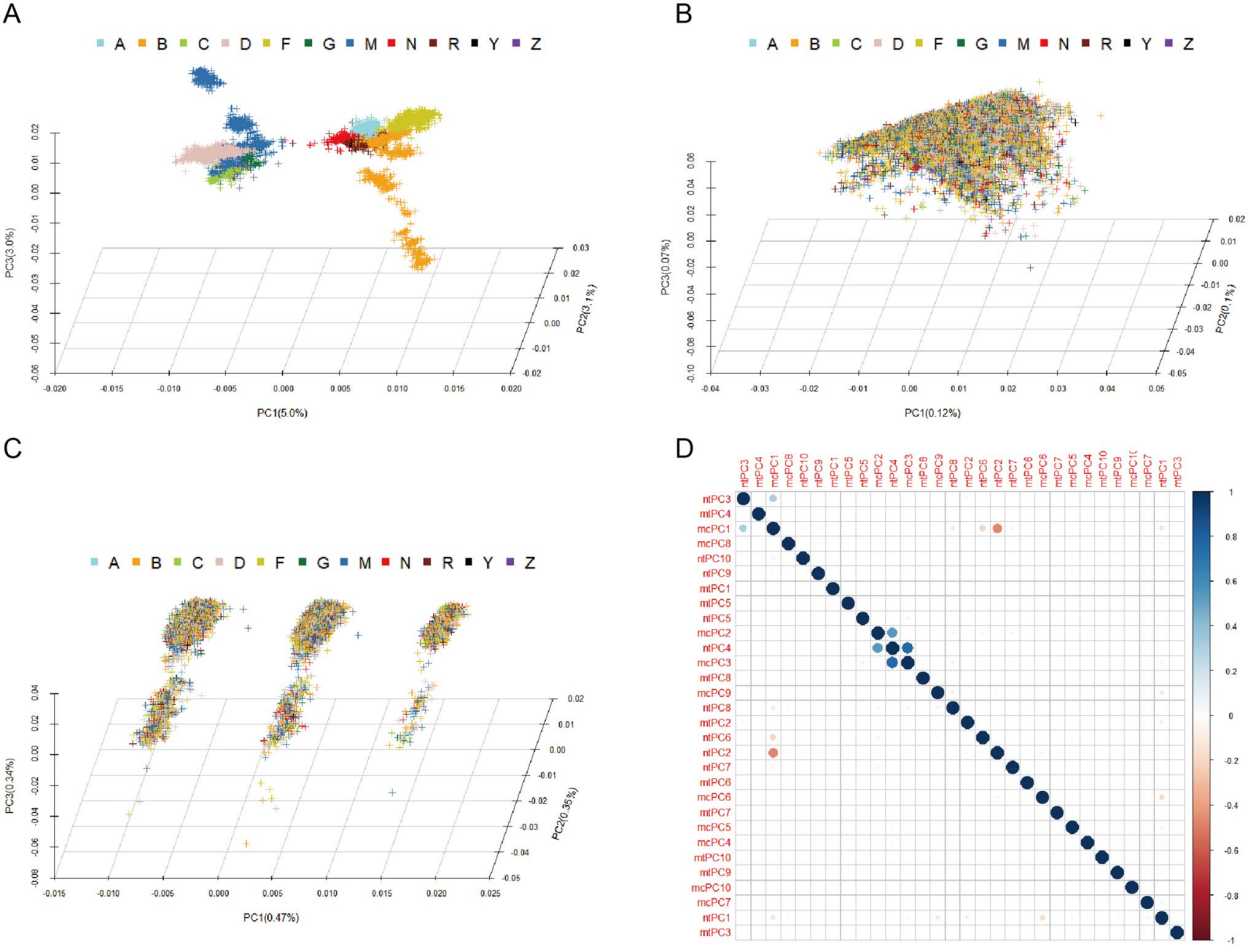
The population structure of mtDNA, ntDNA and mitoCato gene variants. The first three PCA plots of mtSNVs (**A**), ntSNVs (**B**) and variants of mitoCato genes (**C**) showed the genetic structures of each gene sets. **D** The correlation plot of the first ten PCs of each gene set. The abbreviations were as follows: *mt* mitochondria, *nt* nucleu genome, *mc* mitoCato

Being maternally inherited, mitochondrial DNA is thought to be independent of the inheritance of the nuclear genome, even though both mitochondrial and nuclear genomes jointly interact to make mitochondria function well (Wei et al. 2019). To evaluate the potential relationship between the two kinds of genomes, we calculated the principal components of mtDNA, ntDNA and 1136 nuclear genes with mitochondrial protein localization in MitoCarta3 (noted as mcDNA), respectively. 11 haplogroups in the first three mtPCs plot were clustered into the N cluster and the M cluster. The N cluster included A, B, F, N, R and Y haplogroups, and the M cluster included C, D, G, M and Z haplogroups. It showed the consistency between the ancestry assignment and the mtPCs analysis (Fig. 2A). No clear cluster was formed in the first three ntPCs plot indicating that there was no discernible ntDNA population structure (Su et al. 2023), meanwhile, haplogroup tags were mixed together in the plot (Fig. 2B). Three distinct groups appeared in the first three mcDNA with a minimum of 1.16% explainable variance, whereas the haplogroup tags were also intermixed (Fig. 2C). The correlation plot for the first 10 PCs of mtDNA, ntDNA or mcDNA showed a strong correlation of mcPC3-ntPC4 pair, and a weak correlation of mcPC1-ntPC2 pair and mcPC1-ntPC3 pair, while no correlation of mtPC-ntPC pairs or mtPC-mcPC pairs (Fig. 2D). No evidence of the related signals of genetic structure between mitochondrial and nuclear genomes was observed, even though we focused on the nuclear genes encoding mitochondria-located proteins.

### Single variant-based mtDNA association analysis

**Fig. 3:**
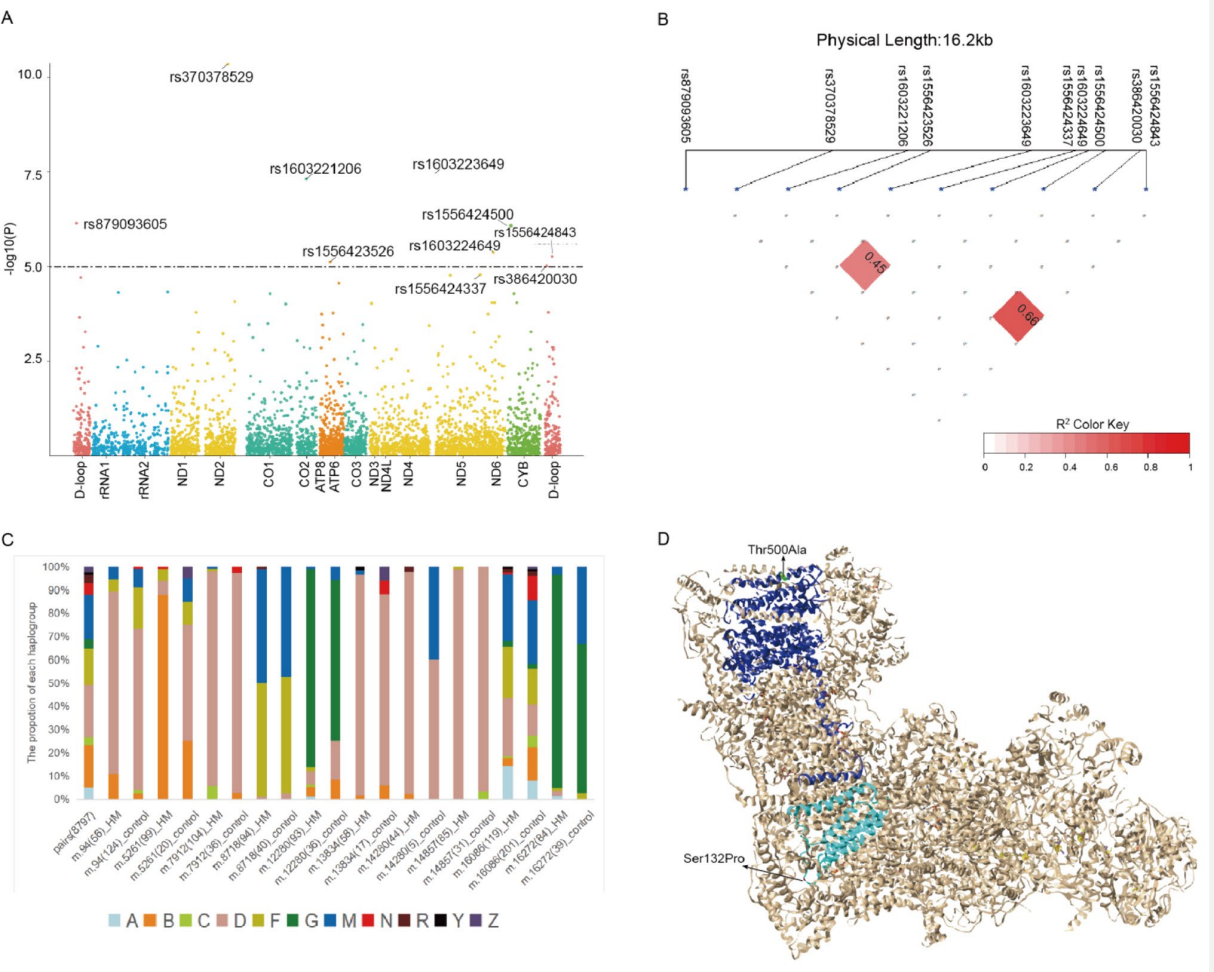
The mtDNA variants associated with high myopia. **A** Mitochondrial regional plots indicating mtDNA-HM association variants detected by the plink_firth model. Nine sites were reaching the corrected *P* at 1.02 × 10^–5^ (bonferroni correction). **B** The linkage disequilibrium (LD) was detected between rs1603223649 and rs1556424843 (*R*^2^ = 0.66), while rs1603221206 and rs1556424337 also showed strong linkage (*R*^2^ = 0.45). **C** The proportion of haplogroups in cases and controls with associated sites. The “pair (8797)” represent the 8797 cases and 8797 controls used in the association study. **D** A human cryo-EM structure of complex I (PDB:5XTD), with ND5 highlighted in blue and ND6 in cyan. Thr500 of ND5 is located in an α helix and its position is colored with green. Ser132 of ND6 is located in a loop and colored with red. All structure visualizations were generated using UCSF Chimera software (v.1.16)

A total of 4904 bi-allele SNPs were included in the whole mitochondria-wide association analysis. The vast majority of these variants (*n* = 4558, 92.9%) were rare variants with allele frequency (AF) of less than 0.5%; while the low-frequency variants (0.5% ≤ AF < 5%) and common variants (AF ≥ 5%) accounted for 5.8% (*n* = 285) and 1.3% (*n* = 61), respectively. Nine variants reached the corrected *P* value of 1.02 × 10^–5^ in all three models (Fig. 3A; Table 2), and the *P* value rank of variants showed a consistent similarity among three models. Just like the deviation from 1 reported in other mtDNA association study (Yonova-Doing et al. 2021), the inflation factor (λ) was 1.43. The frequency of effect alleles is ranging from 0.005 to 0.013, and those variants distributed across several top-haplogroups. The frequency of these variants were queried in MTCards for the Chinese population (Wang et al. 2022) and MITOMAP for the global population (Lott et al. 2013) (Table 2). We observed comparable frequency distribution in MTCards as in controls, showing the consistency of frequency in Chinese populations. The frequencies in six out of the nine variants were lower in MITOMAP than in controls, indicating those variants were specific for the Chinese population.

**Table 2:** The mtDNA variants associated with high myopia

Rs_id	Variant	*P*_firth	*P*_ SW^a^	*P*_ SS^b^	Gene	Annotation	Case^c^ (het, hom)	Control^d^ (het, hom)	OR^e^	MTCards^f^	MITOMAP^g^	Haplogroup	Sub-haplogroups^h^ (case,control)
rs370378529	m.5261G > A	4.38E−11	4.67E−11	5.98E−13	ND2	Synonymous	0.011 (0,99)	0.0023 (0,20)	5.25	0.0015	0.002	M;Z;D;N;F;B	B4b1c (88%, 25%)
rs1603223649	m.12280A > G	3.47E−08	3.61E−08	1.35E−08	tRNA-Leu	–	0.010 (2,91)	0.0041 (0,36)	3.41	0.0037	0.0014	M;C;G;D;A;F;B	G2a4 (85%, 69%)
rs1603221206	m.7912G > A	4.87E−08	5.68E−08	1.77E−08	CO2	Synonymous	0.012 (1,103)	0.0041 (0,36)	3.01	0.0049	0.0011	M;C;D;N;F;B	D4a3b (90%, 91%)
rs879093605	m.94G > A	7.18E−07	4.08E−07	2.12E−07	D-loop	–	0.0064 (0,56)	0.014 (0,124)	0.44	0.015	0.004	M;C;D;N;F;B	D4e1 (66%, 68%)
rs1556424500	m.14857T > C	8.13E−07	8.71E−07	3.08E−07	CYB	Synonymous	0.010 (0,85)	0.0035 (1,30)	2.90	0.0028	0.0009	C;D;F	D4e3 (89%, 74%)
rs1603224649	m.14280A > G	4.17E−06	2.65E−06	1.86E−08	ND6	Missense (Ser132Pro)	0.0049 (1,43)	0.00057 (0,5)	8.43	0.0003	0.0013	M;D;A;R;B	D5a2 (95%, 60%)
rs1556424843	16272A > G	5.41E−06	5.47E−06	3.54E−06	D-loop	–	0.0095 (1,83)	0.0044 (0,39)	2.73	0.0025	0.0027	M;G;D;A;F	G2a4 (92%, 64%)
rs1556423526	m.8718A > G	7.63E−06	6.79E−06	3.72E−06	ATP6	Synonymous	0.011 (1,93)	0.0045 (0,40)	2.37	0.0093	0.0025	M;D;F;B	M71 (49%, 48%), F1a3 (46%, 50%)
rs386420030	m.16086T > C	9.79E−06	8.16E−06	6.53E−06	D-loop	–	0.013 (5,114)	0.022 (14,187)	0.59	0.017	0.023	M;C;Z;G;D;N;Y;A;R;F;B	–
rs1556424337	m.13834A > G	1.62E−06	1.56E−05	5.18E−06	ND5	Missense (Thr500Ala)	0.0066 (0,58)	0.0019 (1,16)	3.41	0.0025	0.0006	M;Z;D;N;Y;B	D4a3b (95%, 82%)

^a^P_SW, the *P* value calculated by the SingleWald method

^b^P_SS, the *P* value calculated by the SingleScore method

^c^The allele frequency in HM, and the HM number of heroplasmy and homoplasmy in the bracket

^d^The allele frequency in control, and the control number of heroplasmy and homoplasmy in the bracket

^e^OR, the odds ratio

^f^MTCards, allele frequency in MTCards database

^g^MITOMAP, allele frequency in MITOMAP database

^h^The major sub-haplogroup harboring the associated variant and its percentage in case and control with the variant in the bracket, respectively

Interestingly, rs1603221206 in *CO2* and rs1556424337 in *ND5* (approaching the significant level of 1.02 × 10^–5^) showed moderate linkage (*R*^2^ = 0.45), while rs1603223649 in tRNA^Leu^ (CUN) and rs1556424843 in D-loop also showed strong linkage (*R*^2^ = 0.66) (Table 2; Fig. 3B). Although nine variants distributed across several haplogroups, the rest of variants had predominant haplogroups excluding m.16086T > C (Fig. 3C). Eight out of nine variants were mostly located in related sub-haplogroups, i.e. m.5261G > A in B4b1c, m.12280A > G in G2a4, m.7912G > A in D4a3b, m.94G > A in D4e1, m.14857T > C in D4e3, m.14280A > G in D5a2, m.16272A > G in G2a4, m.8718A > G in M71 and F1a3 (Table 2), showing that the sub-haplogroup background can increase the susceptible risk for high myopia. The most significant variant, rs370378529 (OR = 5.25), was a synonymous mutation in the NADH-ubiquinone oxidoreductase chain 2 (*ND2*) gene, and distributed among six haplogroups from the M (M; Z; D) cluster to the N (N; F; B) cluster (Table 2; Supplementary Material, Fig. S2). It has the same low frequency in MTCards (Wang et al. 2022) and MITOMAP (Lott et al. 2013) as in controls. The other two genes of complex I, *ND5* and *ND6*, carried two missense mutations rs1556424337 (Thr500Ala in the outside of an α helix of *ND5*) and rs1603224649 (Ser132Pro in a loop of *ND6*) (Fig. 3D). Synonymous mutations being associated with HM were located in *CYB* (rs1556424500), *CO2* (rs1603221206) and *ATP6* (rs1556423526), which consist complex III, IV and V, respectively. The A12280G mutation (rs1603223649) occurred at the dihydrouridine loop of tRNA^Leu^ (CUN) (Supplementary Material, Fig. S4) (Kirino et al. 2006), and widely distributed across from M cluster to N cluster. In addition, two mutations of D-loop, G94A (rs879093605) and T16086C (rs386420030), showed protective effects against HM.

The expression analysis of candidate genes showed high expression of *ND2*, *ND5*, *CO2*, *CYB* and *ATP6* in retina at the transcriptional level (Supplementary Material, Fig. S5), while *ND6* has high expression level in retina at the translational level (https://www.proteomicsdb.org/proteomicsdb/#human/ proteinDetails/P03923/expression).

### MtDNA polygenic risk score (PRS) of HM

**Fig. 4:**
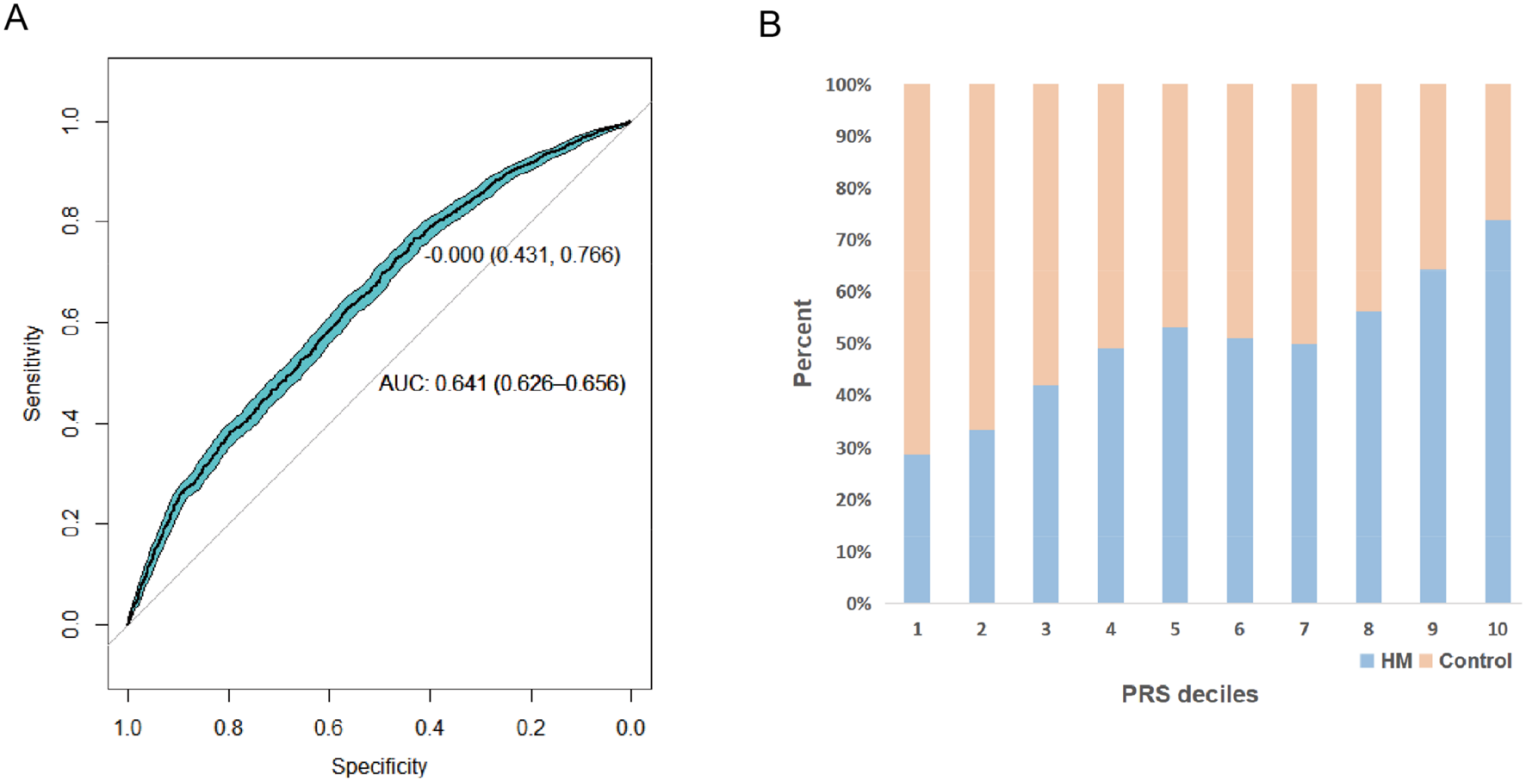
The distribution of polygenic risk score (PRS) in the validation cohort. **A** The AUC plot of polygenic risk score in the validation cohort. The cyan region indicated the 95% confidence interval (CI) of curves with 100 bootstrap. The threshold of AUC at 0.641 is 0 with specificity of 0.431 and sensitivity of 0.766. **B** The decile rank of PRS from low to high in HM and controls

In order to evaluate the potential efficacy of mtDNA variants in detecting HM patients, we utilized the mtDNA to perform PRS analysis in the MAGIC cohort. The receiver operating characteristic (ROC) curve and the corresponding area under the curve (AUC) showed the ability of mitochondria-wide genetic variants for predicting HM at 0.641 (95% CI 0.626, 0.656) with specificity of 0.431 and sensitivity of 0.766 (Fig. 4A). The phenotypic variance explained by the PRS was 8.3% (*R*^2^) in the validation cohort. In comparison with controls, individuals with PRS in the upper percentiles had an increased risk of HM, while individuals with PRS in the lower percentiles had a decreased risk of HM (Fig. 4B). For example, individuals in the top 10% of the PRS distribution had 2.8 times higher odds of HM as compared to controls, and those in the top 20% had 2.3 times; those in the bottom 20% had 0.45 times, and bottom 10% had 0.40 times lower odds of HM when compared to controls (Fig. 4B).

## Discussion

MtDNA association studies were successfully utilized to explore common complex diseases in the UK Biobank using 265 Affymetrix genotyping mtDNA variants, coupled with collected deep phenotypic data of half a million participants (Yonova-Doing et al. 2021). The good performance was also reported in BioBank Japan Project using 206 Illumina genotyping mtDNA variants, coupled with 99 clinical phenotypes of 147,437 Japanese individuals (Yamamoto et al. 2020). In the present study, we performed a sequencing-based cohort study of mitochondria variants in one of the most crucial eye diseases, HM.

First, we conducted a large case–control cohort study in 8,797 ancestry-matched pairs with the sufficient statistical power to detect a mitochondrial variant encoding a relative risk larger than 2 for HM according to the reported power curves of whole mitochondrial genome association studies (McRae et al. 2008; Wang et al. 2010). In order to minimize the effect of population stratification due to maternal inheritance of mitochondria, we annotated individuals into each haplogroup, and matched case–control pairs for each haplogroup. Such a strategy could maximize the statistical power of association study by aggregating large samples. Meanwhile, it is facilitated to measure the relationship of population structure between the mitochondria genome and nuclear genome, and to minimize the effect of nuclear population stratification. Second, in contrast to the nuclear genome (Elson et al. 2001; Yamamoto et al. 2020), high depth of whole-mitochondria genome sequencing allowed us to obtain dense variants rather than sparse tag SNPs in lack of linkage disequilibrium (LD), greatly increasing the possibility to yield causal variants for trait of interests.

In this mtDNA association study, nine mtDNA variants were identified as susceptible loci of HM. These variants were evenly distributed across the N and M clusters, and none of them belonged to a specific haplogroup in the Chinese population (Yao et al. 2002). The most significant variant (rs370378529) in *ND2* along with other two missense variants (rs1556424337 and rs1603224649) in *ND5* and *ND6* affected the function of the core parts in the mitochondrial respiratory chain complex I. The complex I, consisting of the seven mitochondrial-encoded or 38 nuclear-encoded subunits, is the first and largest enzyme of the mitochondrial respiratory chain and oxidative phosphorylation (OXPHOS) system. It is essential in transferring electrons from reduced NADH to coenzyme Q10 (CoQ10, ubiquinone) and in pumping protons to maintain the electrochemical gradient across the inner mitochondrial membrane for complex V (ATP synthase) to synthesize ATP from ADP and inorganic phosphate (Fassone and Rahman 2012). The complex I is also the major site for the generation of reactive oxygen species (ROS), which are important signaling molecules determining the intracellular steady state of the mitochondrial membrane potential, the cell proliferation rate, and apoptosis rate (Tian et al. 2021). The potential mechanisms for rs370378529, rs1556424337 and rs1603224649 to contribute to the development of HM could be their effects on oxidative stress, energy metabolism, or apoptosis. Genetic defects for the complex I deficiency can be mutations in either mitochondrial DNA (mtDNA) or nuclear-encoded structural subunits of the enzyme or mutation of nuclear-encoded complex I assembly factors. Complex I deficiency is clinically heterogeneous from infantile-onset Leigh syndrome, lactic acidosis and stroke-like episodes (MELAS) syndrome, adult-onset encephalomyopathic syndromes to Leber’s hereditary optic neuropathy (LHON) with single eye involvement (Table 1). Whereas *ND2* is also reported to be implicated in LHON with the G5244A mutation (Brown et al. 1995) or the C4640A mutation (Brown et al. 2001). The novel missense mutation: c.798C > G (p. Asp266Glu) in *NDUFAF7*, a nuclear-encoded complex I assembly factor, has been associated with pathogenic myopia by impairing the assembly and stabilization of mitochondrial complex I (Wang et al. 2017). In this study, the two confirmed complex I mutations of LHON in MITOMAP, G11778A and T14484C, were rarely found in HMs and controls with no significant difference in frequency (Table 1). Owing to the functional importance of the complex I and pathogenic severity of its deficiency, the susceptible sites for high myopia should be less conserved, such as synonymous sites or missense sites with less structural change. The two identified missense loci, rs1603224649 and rs1556424337, were both reported to be associated with Leigh syndrome in the ClinVar database with the accession numbers of RCV000855084.1 and RCV000854997.1, respectively. However, their clinical significances were likely-benign and benign, which fitting the mild symptoms of common disease, like HM (Wei et al. 2017; Wei and Chinnery 2020).

Interestingly, eight out of nine variants were predominantly located in related sub-haplogroups, and especially rs1603221206 in *CO2* and rs1556424337 in *ND5* showed strong linkage with the sub-haplogroup D4a3b, while rs1603223649 in tRNA^Leu^ (CUN) and rs1556424843 in D-loop also showed strong linkage within the sub-haplogroup G2a4 (Table 2; Fig. 3B), indicating that the sub-haplogroup background can increase the susceptible risk for high myopia like in the other disease. Although G7912A (rs1603221206) and A13834G (rs1556424337) is 5922 bp apart in mitochondrial genome, the haplotype of G7912A in *CO2* and A13834G in *ND5* was enriched in HMs. The mtDNA had sparse non-distance-dependent linkage disequilibrium decay, and formed haplotypes spanning long distance (Supplementary Material, Fig. S6), which was also observed in the Japanese population (Yamamoto et al. 2020). The LHON susceptibility allele (m.14502T > C, p. 58I > V) in the *ND6* gene modulated the phenotypic expression of primary LHON-associated m.11778G > A mutation in the *ND4* gene, which was demonstrated by 22 Han Chinese pedigrees carrying m.14502T > C and m.11778G > A mutations exhibiting significantly higher penetrance of optic neuropathy than those carrying only m.11778G > A mutation (Jiang et al. 2016). The combination variants of *CO2* (T8241G, p. F219C) and *ND5* (G13276A, p. M314V) were reported in a family with maternally inherited diabetes and deafness (MIDD) associated with retinopathy (Tabebi et al. 2017). Such co-mutations between *CO2* and *ND5* are rare in global population (Verma et al. 2022), suggesting a possible mitochondrial genetic interaction in HM patients. The non-canonical cloverleaf structures of mt tRNAs distinguishing them from cytoplasmic tRNAs are highly susceptible to point mutations, which is a primary cause of mitochondrial dysfunction and are associated with a wide range of pathologies including mitochondrial encephalomyopathies, opthalmoplegia and cardiomyopathy (Suzuki et al. 2011). The A12280G mutation occurring at the dihydrouridine loop of tRNALeu (CUN) was reported to be associated with hypertension in a Chinese family (Lin et al. 2019), meanwhile, it was related to Juvenile myopathy, encephalopathy, lactic acidosis and stoke in the ClinVar database (RCV000851072.1, benign). Such a less conserved mutation may decrease the steady-state level of mt-tRNA in tRNAs metabolism and cause subsequent defects of mitochondrial protein synthesis in associated HM patients.

PRS provides a liability measure of the overall risk of an individual’s genetic susceptibility to disease, which is an integral part of precision medicine (Torkamani et al. 2018). The nuclear-variants-based PRS, derived from 687,289 HapMap3 SNPs from the largest genome-wide association study of myopia in Europeans to-date (*n* = 260,974), was assessed on its ability to predict HM versus controls in an Asian population (Kassam et al. 2022). The results of our study demonstrated that mitochondrial variation could also account for some of the observed variances in HM patients, and mtDNA-based PRS could be a useful adjunctive clinical tool in identifying myopic children at the highest risk for developing HM.

The limitations of this study includes the exclusion of possible haplogroup-sensitive variants in HM using case–control pairs in each top-haplogroup and potential ascertainment bias due to the homogeneity of the study population. Meanwhile, replication studies for these findings are needed, and the integration with other eye mitochondrial omics data will improve our understanding of the genetic basis of HM for future research.

As a summary, we report the first large-scale whole-mitochondrial genome study on high myopia in a Han Chinese population, identifying nine novel mitochondrial genetic variants associated with HM, highlighting the critical role of the mitochondrial respiratory chain complex I in developing HM, and demonstrating the sub-haplogroup background can increase the susceptible risk for high myopia and HM can be predicted with mtDNA variants.

## Supplementary Information

Below is the link to the electronic supplementary material.

Supplementary file1 (DOCX 5682 KB)
