# Supplementary material for: Association of mitochondrial DNA variation with high myopia in a Han Chinese population
Source: Mol Genet Genomics. 2023 Jun 5;298(5):1059–71. doi: 10.1007/s00438-023-02036-y (PMC10363046; doi:10.1007/s00438-023-02036-y)
Supplement: Supplementary file 1 — Supplementary file1 (DOCX 5682 KB) [file 438_2023_2036_MOESM1_ESM.docx]

**
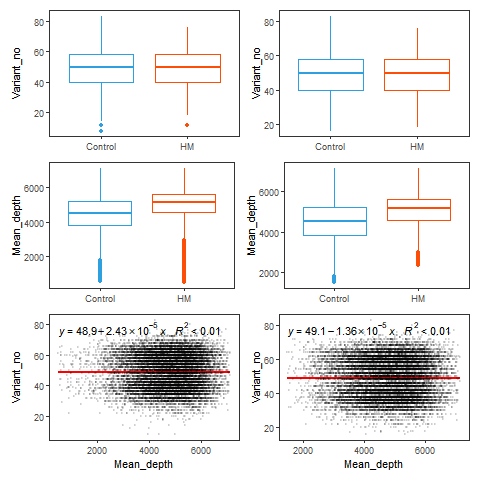
**

**Figure S1. Quality control of samples based on mtDNA sequencing depth and detected variants.** The boxplots of variants being called (A), and mean sequencing depth (B) in each samples of initial HM and the control. (C) The scatterplot between detected variants and mean sequencing depth in each samples of initial HM and the control. The boxplots of variants being called (D), and mean sequencing depth(E) in each samples of HM and the control after removing 132 cases and 39 controls (mean +/- 3SD). (F) The scatterplot between detected variants and mean sequencing depth in each samples of HM and the control after removing outliers.

**
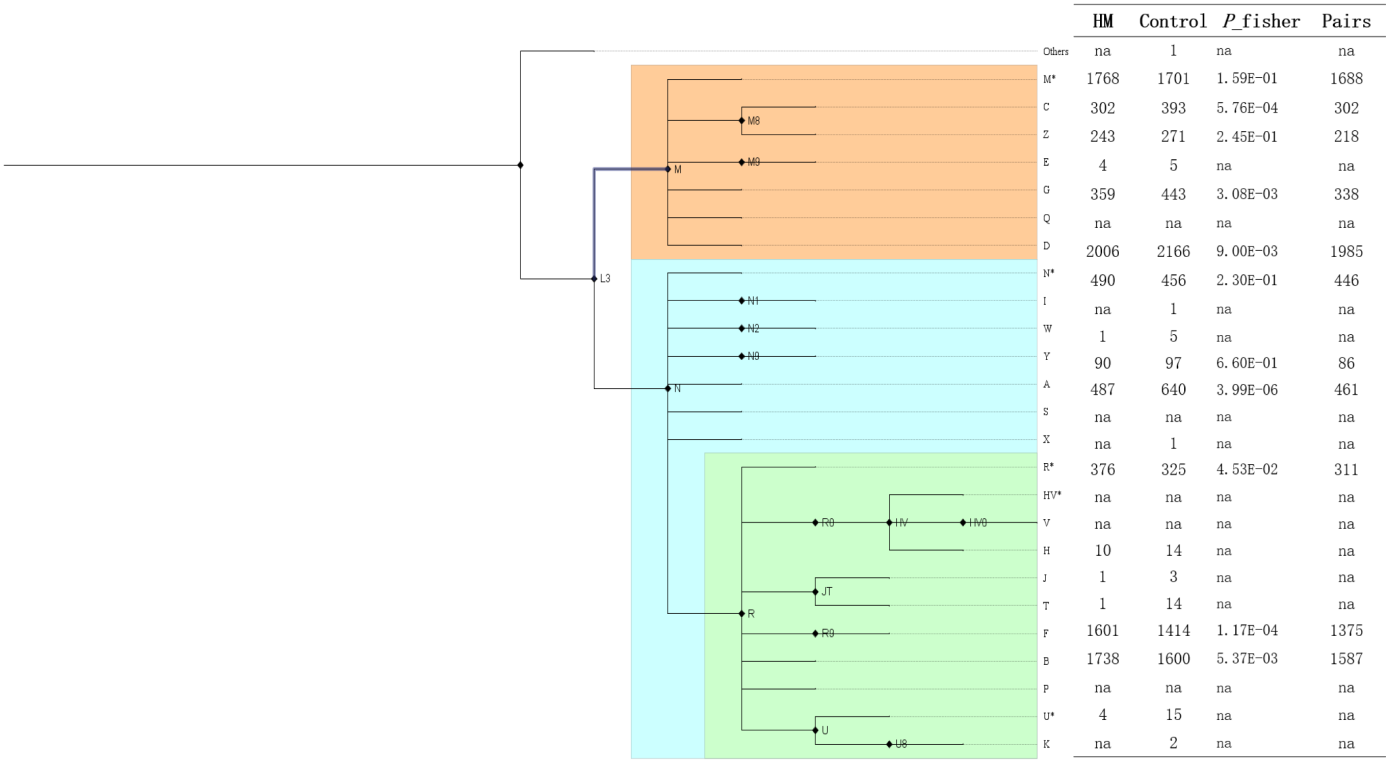
**

**Figure S2. Haplogroup assignment and matched pairs in HM and the control.** The samples in each annotated haplogroups were to do pair matching by pcaMatch. The colors of orange and blue indicates the M cluster and the N cluster, respectively.

**
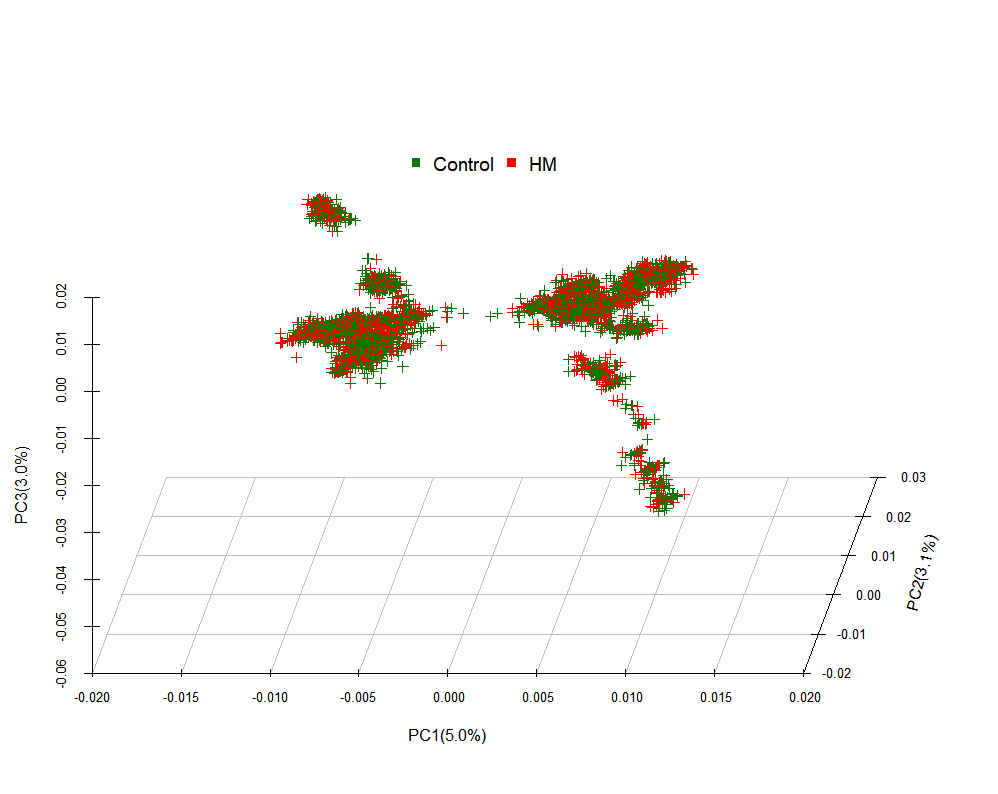
**

**Figure S3. PCA plots of match pairs in HM and the control.**

**
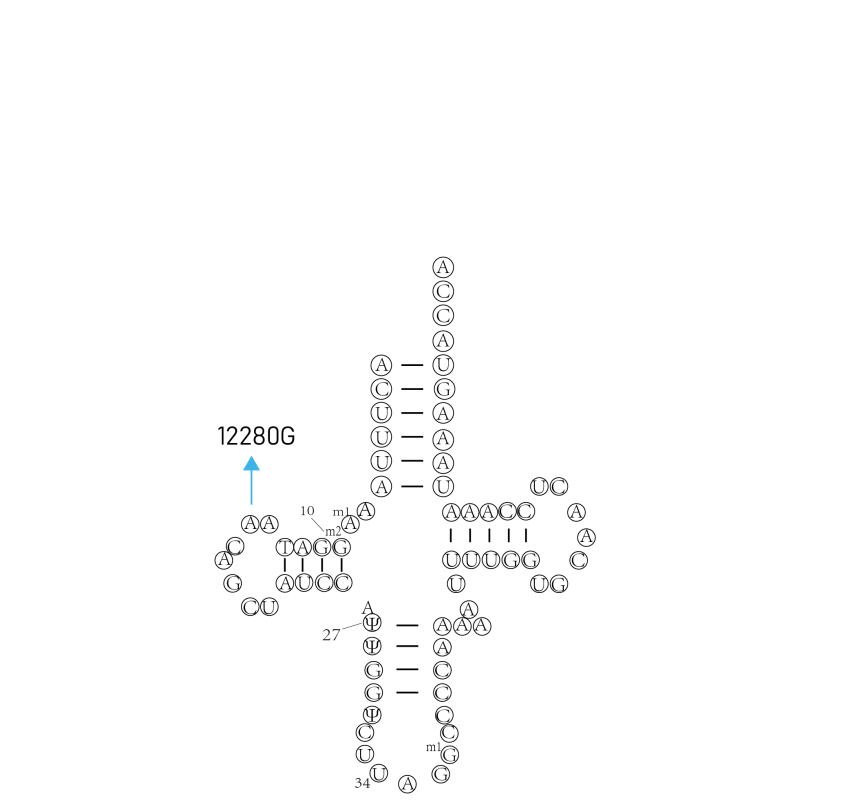
**

**Figure S4.** **The A12280G variant showed in the dihydrouridine loop of tRNA^Leu^ (CUN).**

**
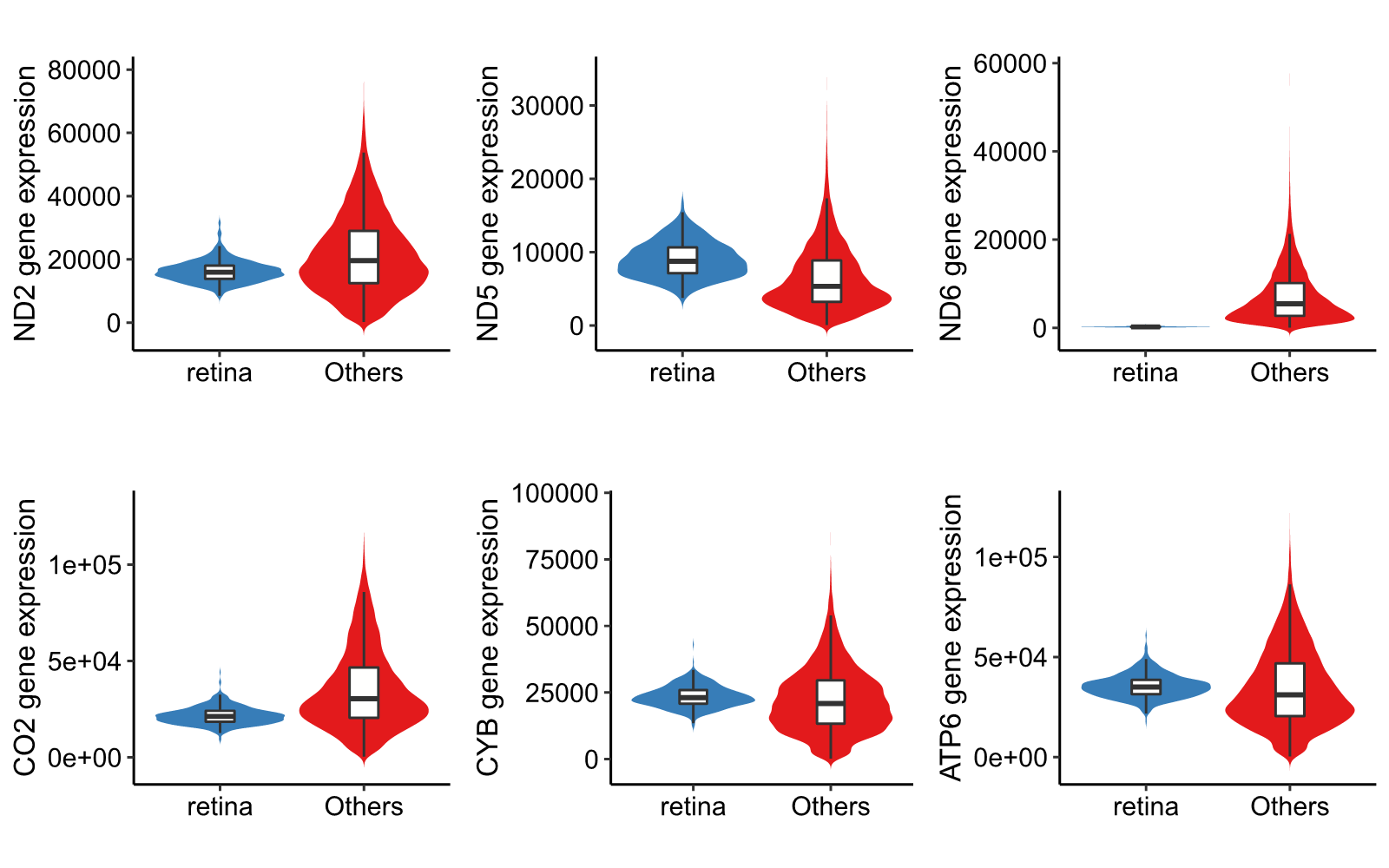
**

**Figure S5. Transcriptional expression of HM-associated mt genes in retina and other tissues.**

**
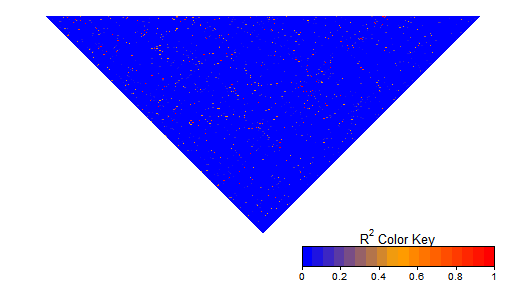
**

**Figure S6. The linkage disequilibrium decay across entire mitochondria genome.** It was calculated by R package “Ldheatmap” using mtDNA variants above minor allele frequency of 0.005 in 8,797 HM and 8,797 controls.
